# Etiologies of fever of unknown origin in HIV/AIDS patients, Hanoi, Vietnam

**DOI:** 10.1186/s12879-022-07049-3

**Published:** 2022-01-18

**Authors:** Thu Kim Nguyen, Yen Hai Nguyen, Hao Thi Nguyen, Quang Minh Khong, Ngoc Kim Tran

**Affiliations:** 1 Hanoi Medical University, Hanoi, Vietnam; 2 National Hospital for Tropical Diseases, Hanoi, Vietnam; 3 ActionAid Vietnam, Hanoi, Vietnam

**Keywords:** Fever of unknown origin, HIV/AIDS patients, CD4 cell count, Vietnam

## Abstract

**Background:**

Fever of unknown origin (FUO) is a challenge for clinicians treating patients with HIV/AIDS. CD4 counts can be helpful in the diagnosis and treatment. This study aimed to determine several common etiologies of FUO stratified by CD4 count levels in HIV/AIDS patients.

**Methods:**

A cross-sectional retrospective and prospective study was conducted in 195 HIV/AIDS patients with FUO admitted to the National Hospital for Tropical Diseases from January 2016 to June 2019. Clinical parameters, immune status, and etiologies for each patient were recorded. Odds ratios were calculated to compare the distributions of common etiologies in groups with two different CD4 count levels: < 50 cells/mm^3^ and ≥ 50 cells/mm^3^.

**Results:**

The proportions of opportunistic infections and noninfectious etiologies were 93.3% and 3.6%, respectively. Tuberculosis was the most common opportunistic infection (46.7%), followed by talaromycosis (29.2%) and Pneumocystis jiroveci (PCP) infection (20.5%). Tuberculosis was predominant in all CD4 level groups. Most patients with talaromycosis had CD4 counts below 50 cells/mm^3^. In total, 53.8% of the patients were infected by one pathogen. The risks of tuberculosis and talaromycosis in FUO-HIV patients were high when their CD4 counts were below 50 cells/mm^3^.

**Conclusions:**

Opportunistic infections, especially tuberculosis, are still the leading cause of FUO in HIV/AIDS patients. Tuberculosis and Talaromyces marneffei (TM) infection should be considered in patients with CD4 cell counts < 50 cells/mm^3^. This study implies that guidelines for appropriate testing to identify the etiology of FUO in HIV/AIDS patient based on the CD4 cell count should be developed, thereby reducing resource waste.

**Supplementary Information:**

The online version contains supplementary material available at 10.1186/s12879-022-07049-3.

## Background

Fever of unknown origin (FUO) is a common manifestation in people living with human immunodeficiency virus (HIV) and acquired immune deficiency syndrome (AIDS), particularly those in an advanced stage [1]. FUO is divided into four groups: (a) classic FUO, defined by Petersdorf and Beeson in 1961; (b) in-hospital FUO; (c) FUO associated with leukopenia and (d) FUO associated with HIV [2]. According to the World Health Organization (WHO), there were 37.7 million people living with HIV and 1.5 million people newly infected with HIV in 2020 globally [3]. The rates of FUO among HIV-infected persons have varied in several previous studies, with a wide range of 3.4–21.0% [4–7]. In Vietnam, HIV/AIDS is a major public health problem and the leading health burden nationwide. In 2020, 213,724 people were living with HIV, and 13,955 people were newly infected with HIV [8].

With the development of testing techniques in recent years, more causes of long-term fever in HIV patients have been identified [9, 10]. Several studies in developing countries indicated that opportunistic infections, such as infections due to *Leishmania* parasites or *Mycobacterium tuberculosis*, led to FUO among people living with HIV/AIDS [1, 9]. In another study conducted in a nation in the southeastern Asia, Thailand, *Mycobacterium* tuberculosis infection was the most frequent infectious etiology, followed by Cryptococcus neoformans and *Pneumocystis jiroveci* infections [10].

The prevalence of the etiology of FUO in HIV-infected patients depends on their immune deficiency status, which is reflected by CD4 cell counts [1]. A previous study reported that 77.0% of FUO-HIV patients had CD4 cell counts less than 100 cells/mm^3^ [4]. In another study, patients with advanced HIV infection whose CD4 cell counts were below 50 cells/mm^3^ were at an increased risk of Mycobacteria avium complex (MAC) infection [11]. Thus, CD4 cell counts could be useful in predicting common pathogens causing FUO in patients infected with HIV. Despite advances in diagnostic methods to identify pathogens, diagnosing pathogens causing FUO in HIV-infected patients remains a challenge.

Although Vietnam has a very high prevalence of HIV infection, information on HIV-related FUO has been limited. Since there is no standard diagnosis for FUO, clinicians often try to diagnose the cause of FUO among HIV patients by ordering every conceivable test, which is wasteful, unnecessary and may lead to a misdirected diagnostic FUO work-up [12, 13]. Using appropriate tests for FUO diagnosis based on the CD4 cell counts may be a focused approach to diagnose the etiologies of FUO among HIV patients, particularly HIV patients in advanced stages [11, 14]. Exploring the etiologies of FUO may find a focused appropriate approach that supports reducing the cost of screening tests, wastes the time of clinicians, and eliminates overdiagnosis and overtreatment of FUO among HIV patients in Vietnam. Therefore, to gain more insightful knowledge about the association between pathogens and immune status in patients with HIV/AIDS in Vietnam, this study aimed to examine several common causes of FUO and investigate the association between some common etiologies of FUO in HIV-infected patients and their immune status.

## Methods

### Study design and setting

A cross-sectional, retrospective and prospective study was performed in the National Hospital for Tropical Diseases from January 2016 to June 2019. Medical records of patients were collected, and the inclusion criteria were as follows: (a) age ≥ 18 years, (b) HIV infection, confirmed by three testing methods approved by the Ministry of Health, and (c) FUO, as defined by Durack and Street in 1991 [2]. According to the definition by Durack and Street (1991), HIV-associated FUO has several criteria, including a temperature of 38.3 °C (101 °F) or higher on multiple examinations; confirmed positive serology for HIV infection; fever with a duration of more than 4 for outpatients or more than 3 days for hospitalized patients; and uncertain diagnosis after 3 days despite appropriate investigation, including at least 2 day's incubation of microbiologic cultures [2]. The exclusion criterion was an in-hospital duration too short to make a diagnosis.

For the retrospective study, all medical records of HIV/AIDS patients hospitalized from January 2016 to June 2018 were collected. There were 170 eligible patients included in the study. For the prospective study, HIV/AIDS patients treated in National Hospital for Tropical Diseases from July 2018 to June 2019 were included. After applying purposive sampling, the total sample size was estimated to be 195 participants. We collected their personal information from their medical records, including (1) reasons for hospitalization, (2) history of antiretroviral therapy (ART) treatment and clinical manifestations, and (3) laboratory test results (routine tests, CD4 cell count, chest X-ray and abdominal ultrasound, specific tests for identifying etiologies, other examinations, and diagnostic tests to support causative diagnoses, and bone marrow aspiration or biopsy if patients had abnormal complete blood count (CBC)).

A part of data was used to publish a paper in the Vietnam Journal of Infectious Disease in June 2020 [15]. In this publication, we selected HIV-infected patients having persistent fever diagnosed of at least one infectious etiology to determine and describe several common opportunistic infections in HIV/AIDS patients. The total number of sample size was 182 participants [15].

### Study definitions and observed methods

FUO was defined as a body temperature over 38.3 °C (101 °F) or higher on several examinations and other criteria by Durack and Street (1991) [2]. A case was defined as an opportunistic infection if there was at least one isolated pathogen. A patient was diagnosed with bloodstream infection if there was at least a positive blood culture and systemic inflammatory response syndrome. A case was defined as a noninfectious condition if there was no isolated pathogen and at least one confirmed noninfectious cause, such as hematologic disease, malignancy, or autoimmune disease. All patients were evaluated for clinical stages and immune responses, and then causes of fever were identified. CD4 cell counts were classified into two groups to investigate their association with the causes of FUO: < 50 cells/mm^3^ and ≥ 50 cells/mm^3^. The cutoff point of the CD4 cell count was 50 cells/mm^3^ because a CD4 cell count < 50 cells/mm^3^ is associated with a particularly poor short-term prognosis and is the point at which the fatality rate among HIV/AIDS patients increases, which an important status for the rapid development of infectious diseases [16].

Appropriate diagnostic investigations were based on clinical presentations and initial laboratory and radiographic data. HIV status was confirmed by three positive tests, including enzyme-linked immunosorbent assay (ELISA), SD Bioline HIV 1/2, and Vikia HIV 1/2. The etiological detections were performed according to the standard testing procedures of the National Hospital for Tropical Diseases. The etiologies causing FUO consisted of opportunistic infections and noninfectious causes.

### Diagnostic procedure of FUO etiology

*Tuberculosis (TB)* is a disease caused by Mycobacterium tuberculosis that should be suspected in persons who have the symptoms of TB, such as a persistent and productive cough, which may be bloody, that lasts more than 3 weeks, weight loss, night sweats, a high temperature, and tiredness and fatigue. A definitive diagnosis is made when TB bacteria are isolated from sputum, bronchial fluid, gastric juice, cerebrospinal fluid, pleural fluid, peritoneal fluid, etc. by an acid-fast bacilli (AFB) test, PCR, GeneXpert sequencing, or blood cultures positive for TB.

*Talaromyces Marneffei* (TM) infection has several clinical manifestations, including fever, skin papules, anemia, hepatosplenomegaly, and lymphadenopathy. A definitive diagnosis is made when TM is isolated from skin lesions, blood, or lymph nodes by endoscopy or culture on Sabouraud's medium.

*Pneumocystis jiroveci infection* (PCP) infection manifestations include cough, gradually increasing dyspnea, fever, and night sweats. Chest radiography will show bilateral diffuse interstitial infiltrates, and a bronchoalveolar lavage test will involve Giemsa stain or PCR for PCP or PCP-specific treatment response.

*Bacterial pneumonia* symptoms include a high fever of 39–40 °C, chills, chest pain, cough, and shortness of breath. On examination, there will be consolidation syndrome, auscultation of the lungs with moist rales, and crackles on the affected side. Subclinical factors include white blood cell (WBC) of > 10 G/l, neutrophils > 75%, elevated C-reactive protein (CRP), and elevated procalcitonin (PCT); sputum culture will be positive for bacteria. Chest X-ray and computed tomography (CT) will show triangular opacities at the hilum side, basal outside or bronchial-shaped opacities, possibly at the costophrenic angle.

*Cytomegalovirus (CMV) infection* manifestations include retinitis with blurred vision, moving black spots or spots, dark spots in front of the eyes, photophobia, progression to retinal detachment and blindness if left untreated. It can be affected one eye or both eyes. Retinal damage is usually irreversible. Ophthalmoscopy will show white necrosis in the retina, possibly with isolated retinal hemorrhage or diffuse clusters. A definitive diagnosis includes clinical and PCR positivity for CMV (blood) or viral quantification.

*Septicemia* is diagnosed when 2/4 of the Systemic Inflammatory Response Syndrome (SIRS) criteria are present, there is evidence of infection (infectious syndrome, elevated CRP and/or PCT), organ dysfunction, and the patient responds to antibiotic therapy.

*Toxoplasmosis* clinical manifestations include fever, skin papules, anemia, hepatosplenomegaly, lymphadenopathy. A definitive diagnosis is made after isolation of PM from skin lesions, blood, or lymph nodes by endoscopy and culture on Sabouraud's medium.

*Cryptococcosis* manifestations include any of the following: (1) fungal infection: fever, necrotic papule skin lesions, and pulmonary infiltrates; or (2) meningitis: headache, photophobia, meningoencephalitis, confusion, focal neurological signs, and fever. A definitive diagnosis is made when the fungus isolated from blood or cerebrospinal fluid samples, lymph nodes, or the skin by endoscopy and culture.

*Mycobacteria avium complex (MAC) infection* manifestations include persistent or recurrent fever, weight loss, fatigue, anemia, swollen lymph nodes, diarrhoea, and shortness of breath. A definitive diagnosis is based on isolating MAC from blood or other tissues. Clinicals should consider a diagnosis of MAC if the patient has not responded to TB treatment after 2 to 4 weeks.

*Hemophagocytic lymphohistiocytosis (HLH) syndrome* diagnostic criteria are as follows: (1) confirmation of an HLH-associated genetic mutation; (2) 5 out of 8 of the following signs: fever; enlarged spleen; a decrease in at least 2 out of 3 peripheral blood parameters (Hb 90 g/l; platelets < 100 × 109/L; granulocytes < 1.0 × 109/l); hypertriglyceridemia and/or hypofibrinogenemia (triglycerides ≥ 3 mmol/l; fibrinogen ≤ 1.5 g/l; a myelogram, splenogram or lymph node chart with hematopoietic phagocytosis (no evidence of cell malignancy); a decrease in or loss of NK cell activity; ferritin ≥ 500 mcg/l; and soluble CD25 ≥ 2400 U/ml.

*Non-Hodgkin Lymphoma* "B" symptoms consist of fever, sweating, weight loss, lymphadenopathy, hepatosplenomegaly, possible anemia, and hemorrhage. A definitive diagnosis is based on lymphoma tests, including lymph node biopsy and immunohistochemistry of specific biopsy fragments for lymphoma.

### Data analysis and statistical methods

To address the aims of the study, both descriptive and analytical statistics were analyzed by using SPSS software version 20.0. Qualitative variables are presented as frequencies and percentages (%), while quantitative variables are presented as means and standard deviations (SDs). Comparisons between categorical variables were tested by chi-square and Fisher’s exact tests. Crude odds ratios (ORs) and 95% confident intervals (95% CIs) were used to determine several common etiologies causing FUO among HIV-infected patients stratified by CD4 count.

A multivariate analysis was also performed to determine the independent etiologies of FUO, including comparisons of tuberculosis (yes/no), talaromycosis (yes/no), PCP infection (yes/no) bacterial pneumonia (yes/no), and coinfection (> 2 pathogens, 1–2 pathogen(s)). A p value (p) < 0.05 was considered statistically significant.

## Results

The majority of participants were males, accounting for 75.4% (Table 1). The male/female ratio was 3:1. The mean age was 37.5 years (SD: ± 8.5 years), and half of the total study subjects were of working age (31–40 years). The mean of duration was 4.3 ± 2.2 weeks. At presentation of FUO, 23 patients (11.8%) had stage I–III disease, while 172 patients (88.2%) had stage IV disease. The median CD4 T cell count was 19 cells/mm^3^, with a wide range from 1 to 713 cells/mm^3^ (IQR = 8; 50). A total of 35.9% of the patients were currently on ART.

**Table 1:** Characteristics of participants (N = 195)

Characteristics	n (%) or Mean ± sd
Gender (%)	
Men	147 (75.4)
Women	48 (24.6)
Age (mean ± sd; years)	37.5 ± 8.5
Duration of fever (mean ± sd; weeks)	4.3 ± 2.2
Clinical stages	
Stage I–III (%)	23 (11.8)
Stage IV (%)	172 (88.2)
CD4 count (mean ± sd; cells/mm^3^)	49.7 ± 99.3
< 50 (%)	145 (74.4)
≥ 50 (%)	50 (25.6)
Currently on ART (%)	
No	125 (64.1)
Yes	70 (35.9)

Opportunistic infections occurred in 93.3% of 195 HIV-infected patients with FUO (Table 2). Tuberculosis was the most common opportunistic infection (46.7%), followed by TM infection (29.2%) and PCP infection (20.5%). Less common opportunistic infections included bacterial pneumonia (11.3%), CMV infection (10.3%), sepsis (10.3%), toxoplasmosis (5.6%), Cryptococcus infection (2.6%) and MAC infection (1.0%). There were 7 cases with noninfectious causes (3.6%) and 6 undefined cases (3.1%). Regarding coinfection, 53.8% of the patients were infected by one pathogen, followed by two pathogens (38.0%) and three pathogens (8.2%).

**Table 2:** Prevalence of etiologies among FUO-HIV patients

Etiologies	n (%)
Total	195 (100)
Opportunistic infections	183 (93.9)
Tuberculosis	91 (46.7)
Talaromyces Marneffei (TM)	57 (29.2)
Pneumocystis jiroveci infection (PCP)	40 (20.5)
Bacterial pneumonia	22 (11.3)
Cytomegalovirus (CMV)	20 (10.3)
Septicemia	20 (10.3)
Toxoplasmosis	11 (5.6)
Cryptococcosis	5 (2.6)
Mycobacteria avium complex infection (MAC)	2 (1.0)
Non-infectious causes	7 (3.6)
Hemophagocytic lymphohistiocytosis (HLH) syndrome	4 (2.1)
Non-Hodgkin Lymphoma	3 (1.5)
Undefined causes	6 (3.1)
Coinfection	
1 Pathogen	105 (53.8)
2 Pathogens	74 (38.0)
3 Pathogens	16 (8.2)

Table 3 demonstrates the prevalence of the etiologies by CD4 counts. Tuberculosis was predominant in all subgroups stratified by CD4 counts. Most patients with other opportunistic infections (talaromycosis, PCP infection, bacterial pneumonia, septicemia, CMV infection, and toxoplasmosis) had CD4 counts < 50 cells/mm^3^. In addition, a CD4 count < 50 cells/mm^3^ was reported in all cases of Cryptococcus and MAC infections. The differences in some opportunistic infections, such as tuberculosis, talaromycosis, and septicemia, between the two CD4 count groups were statistically significant. Additionally, there was no significant difference between the number of pathogens involved in coinfections between the CD4 count groups.

**Table 3:** Prevalence of etiologies by levels of CD4 cells count

	Levels of CD4 count		p-value
	< 50 cells/mm^3^	≥ 50 cells/mm^3^	
	n (%)	n (%)	
Total	145 (100)	50 (100)	
Opportunistic infections	138 (95.2)	45 (90.0)	0.19
Tuberculosis	60 (41.4)	31 (62.0)	0.012
Talaromyces Marneffei	48 (33.1)	9 (18.0)	0.048
PCP infection	30 (20.7)	10 (20.0)	0.92
Bacterial pneumonia	18 (12.4)	4 (8.0)	0.60
Septicemia	19 (13.1)	1 (2.0)	0.03
Cytomegalovirus	16 (11.0)	4 (0.1)	0.54
Cryptococcosis	5 (3.5)	0 (0.0)	0.33
MAC infection	2 (1.4)	0 (0.0)	1.00
Toxoplasmosis	7 (4.8)	4 (8.0)	0.48
Non-infectious causes	3 (2.1)	4 (8.0)	0.05
Hemophagocytic Lymphohistiocytosis (HLH) Syndrome	1 (0.7)	3 (6.0)	0.05
Non-Hodgkin Lymphoma	2 (1.4)	1 (50.0)	0.04
Coinfection			
1 Pathogen	79 (54.5)	26 (52.0)	0.72
2 Pathogens	53 (36.5)	21 (42.0)	
3 Pathogens	13 (9.0)	3 (6.0)	

Table 4 shows the crude ORs for several opportunistic infections and coinfections by CD4 count group. Compared to patients with CD4 counts ≥ 50 cells/mm^3^, those with CD4 counts < 50 cells/mm^3^ were 0.43 (95% CI: 0.22, 0.84; p value = 0.01) times less likely to be infected with tuberculosis. On the other hand, individuals with CD4 counts < 50 cells/mm^3^ were 2.25 (95% CI: 1.01, 5.02; p value = 0.04) times more likely to have talaromycosis than those with CD4 counts ≥ 50 cells/mm^3^. These differences were statistically significant. For PCP, bacterial pneumonia, and coinfections (> 2 pathogens), there was no significant difference between the two CD4 cell count groups. However, in the multivariate analysis, individuals with CD4 counts < 50 cells/mm^3^ were 2.88 (95% CI: 1.41, 5.89; p value < 0.01) times more likely to develop tuberculosis.

**Table 4:** Univariate and multivariate analyses of the CD4 counts level among HIV-infected patients with fever of unknown origin at National Hospital for Tropical Diseases

Variables	Univariate		Multivariate	
	OR (95% CI)	p-value	OR (95% CI)	p-value
Tuberculosis	0.43 (0.22, 0.84)	0.01	2.88 (1.41, 5.89)	< 0.01
Talaromycosis	2.25 (1.01, 5.02)	0.04	0.66 (0.25, 1.73)	0.40
PCP infection	1.04 (0.41, 2.32)	1.00	1.15 (0.45, 2.95)	0.78
Bacterial pneumonia	1.63 (0.52, 5.07)	0.39	0.64 (0.19, 2.21)	0.48
Coinfection (> 2 pathogens)	1.10 (0.58, 2.10)	0.76	0.77 (0.34, 1.72)	0.53

## Discussion

In this study, opportunistic infections frequently occurred among HIV patients with FUO, and tuberculosis was the most common, followed by TM and PCP infections. The majority of FUO-HIV patients were infected by a pathogen. In addition, the differences in some opportunistic infections (tuberculosis, TM, septicemia) and noninfections (non-Hodgkin lymphoma) between the two CD4 count groups were statistically significant. The risk of tuberculosis and TM infection was high in FUO-HIV patients with CD4 cell counts < 50 cells/mm^3^.

Among 195 HIV-infected patients with FUO, the percentage of males was three times higher than that of females (75.4% and 24.6%, respectively). This result was similar to the results of a study in Thailand (61.0% and 39.0%, respectively) [10], and a previous study in Vietnam (76.8% and 23.2%, respectively) [17] and our previous study (76.4% and 23.6%, respectively) [15].

FUO is a common symptom in HIV-infected people, especially those with advanced disease. In this study, the median CD4 cell count was 19 cells/mm^3^, with an interquartile range (IQR) of 8–50. The lowest CD4 count was 1 cell/mm^3^, and the highest CD4 count was 713 cells/mm^3^. Approximately 74.3% of the patients had CD4 counts below 50 cells/mm^3^, and the majority of patients had clinical stage IV disease (88.2%). A study in HIV-infected patients with FUO in Thailand also showed that the average CD4 count was 56 cells/mm^3^ [11] which was similar to that in our study (49.7 cells/mm^3^). In addition, the study indicated that the median cell count was 30 cells/mm^3^, the highest count was 500 cells/mm^3^, and the lowest count 0 cells/mm^3^ [11]. Thus, HIV-infected patients with FUO usually have serious immunodeficiency status.

Fever was primarily related to opportunistic infection and sometimes malignancy. The development of opportunistic infection depends on the immunodeficiency status of HIV-infected people, local circulating diseases, and primary and secondary prophylaxis [18]. This study identified that infectious etiologies accounted for the highest proportion (93.3%), which was similar to that in a study performed in HIV-infected patients with FUO in the US (2007), which reported an opportunistic infection proportion of 90.6% [19]. Another study conducted in Thailand showed that 71/90 HIV-infected patients (78.9%) had FUO, 70/71 patients (98.6%) had an infectious etiology, and 1/71 patients (1.4%) had an noninfectious etiology [20].

Among the infectious etiologies of FUO in HIV-infected patients, tuberculosis accounted for the highest proportion, followed by talaromycosis and PCP infection; less common causes consisted of bacterial pneumonia, CMV infection, sepsis, toxoplasmosis, and Cryptococcus infection. The prevalence of tuberculosis in the study was similar to our previous study [15] as well as worldwide [10, 14]. This result may be explained by the fact that HIV injures the body's immune system through CD4 and T-CD8 cells and macrophages, thus allowing tuberculosis bacteria to survive, reactivating latent tuberculosis and allowing the dissemination of tuberculosis bacteria to a greater extent than that of other organisms [21]. This finding indicated that patients (60 individuals) with CD4 counts below 50 cells/mm^3^ accounted for the highest proportion. The difference in the incidence of tuberculosis was statistically significant between the two of CD4 cell count groups. This finding indicates that individuals with CD4 cell counts < 50 cell/mm^3^ are at increased risk of tuberculosis.

The prevalence of Talaromyces infection in our study (29.2%) was greater than that in a study in northern Thailand (17.8%) [20]. Talaromyces fungi grow in tropical regions, especially in Southeast Asia, where the climate is hot and humid. In addition to humans, bamboo rats, which are also found in this area, are natural hosts for TM [22]. The univariate analysis showed that FUO-HIV patients with CD4 cell counts < 50 cell/mm^3^ were at an increased risk of TM. The difference in the incidence of TM infection was statistically significant between the two groups. This result was similar to that of a study in Thailand [10]. According to the Ministry of Health classification of CD4 cell counts, most patients were in the AIDS stage. This finding reflects the clinical manifestation of patients in the study.

PCP-associated pneumonia was the third most common etiology, which was similar to that in Thailand (13.0%) [10] and in the US (13.0%) [9]. The findings showed that PCP infection was a common cause of FUO and a common cause of pneumonia in HIV-infected patients. Therefore, in HIV-infected patients with FUO who had respiratory diseases, the two most common causes were tuberculosis and PCP.

Another finding of the study showed that CMV was an uncommon infectious cause of FUO in HIV-infected patients, which was similar to that of a previous study [18]. CMV infection is a type of opportunistic infection, and the rate varies by sex, age, socioeconomic status, and geographic location [23, 24]. Worldwide, the lowest rate of CMV infection is in Western Europe and the US, and the highest is in South America, Africa, and Asia, often affected nonwhite ethnic minorities and in low-income countries [24].

No patients in the study with Cryptococcus infection, CMV infection, sepsis, toxoplasmosis, or MAC infection had CD4 counts > 100 cells/mm^3^ (data not shown). In Pertel’s study, CMV infection was more common in patients with CD4 counts > 100 cells/mm^3^, and the risk of CMV infection increased when the CD4 count dropped below 100 cells/mm^3^ and increased significantly when the CD4 count was < 50 cells/mm^3^ [25]. All patients infected with Cryptococcus belonged to the group with CD4 counts < 50 cells/mm^3^. This result was different from that in Thailand, in which the number of patients with CD4 counts < 50 cells/mm^3^ was 1.16 times higher than that of patients with CD4 counts > 50 cells/mm^3^ [11]. According to Renold and colleagues, 75.0% of brain toxoplasmosis patients have CD4 counts < 100 cells/mm^3^, and the average CD4 count was 78 cells/mm^3^ [26]. Thus, CMV and Cryptococcus infections are opportunistic conditions occurring during the period of severe immunodeficiency, commonly in patients with a CD4 count < 50 cells/mm^3^.

Regarding noninfectious etiologies, non-Hodgkin lymphoma has been a commonly mentioned cause in studies. In this study, the proportion of non-Hodgkin lymphoma patients (1.5%) was lower than those in Jose Mayo’s study (4.2%) [27] and Danai Kitkungvan’s study (4.0%) [10]. In addition, the study also noted that HLH was a noninfectious cause of FUO in HIV patients. Non-Hodgkin lymphoma was found in patients in both CD4 count groups.

In severe immunodeficiency HIV patients with very low CD4 counts, FUO might be caused by two or more concomitant etiologies. In the study, the majority of patients had a single etiology (53.8%), followed by two and three etiologies (38.0% and 8.2%, respectively); this differed from the results of other studies. In a study in Thailand, 73.6% of the patients had a single infectious etiology, and 26% of patients had 2 or more etiologies [10]. According to a study by Romanee Chaiwarith, 81.7% of patients had a single etiology, and 18.3% had 2 or more concurrent etiologies [20]. Another study in Spain showed that 84.2% of patients had a single etiology, 12.5% of patients had 2 etiologies and 3.3% of patients had 3 or more concomitant etiologies [5]. In Bissuel’s study, 83.7% of patients had a single etiology, and 16.3% had 2 or more etiologies [4]. In Lambertucci’s study, 81.8% of patients had a single etiology [28]. However, these findings indicated that there was no difference between the number of concurrent etiologies and the two CD4 levels.

This study has some limitations. First, the retrospective design might have resulted in insufficient data that might be missing clinical information needed for the aims of the study. Thus, the combination of a prospective study and the application of purposive sampling may be sufficient to obtain a comprehensive picture of the studied issue. Second, since the study was conducted at only one hospital in the northern region of Vietnam, participants might not represent the general population in Vietnam. Additionally, data on the numbers of several other opportunistic diseases and CD4 cell counts in the National Hospital for Tropical Diseases was insufficient to utilize univariate and multivariate analyses. Further studies with larger sample sizes should be performed at more hospitals in other regions of Vietnam (Additional file 1).

## Conclusion

In a study of 195 cases of FUO in HIV/AIDS patients in Vietnam, opportunistic infections, especially tuberculosis, were the leading cause of FUO in HIV/AIDS patients. Screening for tuberculosis and TM infection should be recommended for patients with CD4 counts < 50 cells/mm3. Since most of the identified etiologies causing FUO were diagnosable and treatable, this study implies that guidelines and recommendations for the application of appropriate screening tests to diagnose FUO etiologies in HIV-infected patients based on CD4 counts should be developed to prevent the waste of resources and provide treatment for FUO-HIV individuals.

### Supplementary Information

**Additional file 1.** The data of HIV/AIDS patients in the National Hospital for Tropical Diseases.

## Data Availability

The datasets used and analyzed during the current study are available from the corresponding author on reasonable request.

## Abbreviations

ART: Antiretroviral therapy
AIDS: Acquired immune deficiency syndrome
CBC: Complete blood count
CMV: Cytomegalovirus
FUO: Fever of unknown origin
HLH: Hemophagocytic lymphohistiocytosis
HIV: Human immunodeficiency virus
IQR: The interquartile range
MAC: Mycobacteria avium complex infection
PCP: Pneumocystis jiroveci infection
TM: Talaromyces Marneffei
