# Correction to: Etiologies of fever of unknown origin in HIV/AIDS patients, Hanoi, Vietnam

**DOI:** 10.1186/s12879-024-09223-1

**Published:** 2024-04-15

**Authors:** Thu Kim Nguyen, Yen Hai Nguyen, Hao Thi Nguyen, Quang Minh Khong, Ngoc Kim Tran

**Affiliations:** 1 Hanoi Medical University, Hanoi, Vietnam; 2 National Hospital for Tropical Diseases, Hanoi, Vietnam; 3 ActionAid Vietnam, Hanoi, Vietnam

**Correction: BMC Infectious Diseases (2022)22:61**

10.1186/s12879-022-07049-3

After the publication of this article [1], it came to our attention that some of the data presented had been published previously [2]. In this study [2], HIV-infected patients having persistent fever diagnosed of at least one infectious etiology were selected to determine and describe several common opportunistic infections in HIV/AIDS patients. The total number of participants was 182. This information along with the relevant citation has now been added to the updated version.

In our Discussion section the sentence

This result was similar to the results of a study in Thailand (61.0% and 39.0%, respectively) [10], and a previous study in Vietnam (76.8% and 23.2%, respectively) [16].

should read:

This result was similar to the results of a study in Thailand (61.0% and 39.0%, respectively) (10), a previous study in Vietnam (76.8% and 23.2%, respectively) (17) and our previous study (76.4% and 23.6%, respectively) (15).

Also in our Discussion section the sentence.

“The prevalence of tuberculosis in the study was similar to those in some studies in regional countries as well as worldwide [10, 14].

should read:

“The predominance of tuberculosis in the study was similar to our previous study (15) as well as other studies in the world (10, 14).“

In the reference list the following reference should appear as reference 15: Nguyen, KT, Nguyen, HY, & Pham, NT (2020). Common causes of infection in HIV/AIDS patients with prolonger fever (January 2016 - June 2019). Vietnam Journal of Infectious Diseases, 2 (30), 89–94. 10.59873/vjid.v2i30.166

The original article was updated.

## References

[1] Nguyen TK, Nguyen YH, Nguyen HT (2022). Etiologies of fever of unknown origin in HIV/AIDS patients, Hanoi, Vietnam. BMC Infect Dis.

[2] Nguyen KT, Nguyen HY, Pham NT (2020). Common causes of infection in HIV/AIDS patients with prolonger fever (January 2016 - June 2019). Vietnam J Infect Dis.

